# A rising tide of parasite transcriptomics propels pathogen biology

**DOI:** 10.1371/journal.pbio.3001997

**Published:** 2023-01-25

**Authors:** Manoj T. Duraisingh, Marc-Jan Gubbels, Kourosh Zarringhalam

**Affiliations:** 1 Department of Immunology & Infectious Diseases, Harvard TH Chan School of Public Health, Boston, Massachusetts, United States of America; 2 Department of Biology, Boston College, Chestnut Hill, Massachusetts, United States of America; 3 Department of Mathematics, University of Massachusetts, Boston, Massachusetts, United States of America; 4 Center for Personalized Cancer Therapy, University of Massachusetts, Boston, Massachusetts, United States of America

## Abstract

Twenty years ago, the first transcriptome of the intraerythrocytic developmental cycle of the malaria parasite Plasmodium falciparum was published in PLOS Biology. This Perspective looks at how transcriptomics studies have transformed the study of parasite biology in the years following its publication.

## Teaser

Twenty years ago, the first transcriptome of the intraerythrocytic developmental cycle of the malaria parasite *Plasmodium falciparum* was published in *PLOS Biology*. Since then, transcriptomics studies have transformed the study of parasite biology.

This article is part of the *PLOS Biology* 20th Anniversary Collection.

Infection of metazoans by eukaryotic pathogens is a major source of disease, including in humans. These pathogens range from single-celled protozoans to multicellular worms, covering over a billion years of evolution. Parasites exhibit striking changes in morphology as they progress through their complex life cycles and the varied environmental niches they inhabit. These waves of proliferation and differentiation are powered by the regulation of gene expression. Understanding parasite biology through the lens of gene regulation is fundamental for translation to therapeutics, including approaches for directly targeting the RNA as well as the essential enzymes responsible for their synthesis and turnover.

**Fig 1:**
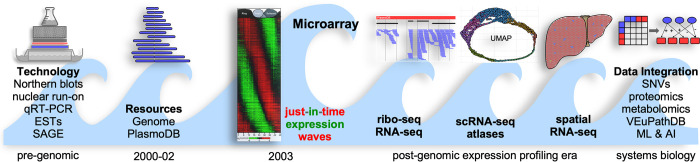
The evolution of parasite transcriptomics. The advent of the expression microarray was a transformative event that for the first time revealed dynamic, genome-wide expression changes. Over time, in the postgenomic era, this has become more refined in spatiotemporal resolution. The next wave of systems biology requires new computational tools that are expected to reveal new aspects of parasite biology for translation. Adapted from [1]. AI, artificial intelligence; EST, expressed sequence tag; ML, machine learning; qRT-PCR, quantitative reverse transcription PCR; ribo-seq, ribosome sequencing; RNA-Seq, RNA sequencing; SAGE, Serial Analysis of Gene Expression; scRNA-seq, single-cell RNA sequencing; SNV, single-nucleotide variation; UMAP, Uniform Manifold Approximation and Projection.

The advent of microarrays in the late 1990s heralded the age of transcriptomics in model systems. Twenty years ago, in a landmark paper published in the first issue of *PLOS Biology* [1], Bozdech, Llinas, and colleagues described the first acquisition of the transcriptome of the intraerythrocytic developmental cycle (IDC) of the apicomplexan malaria parasite *Plasmodium falciparum*, which still infects over 250 million people and kills about 500,000 people each year. This work was monumental, particularly considering that the *P*. *falciparum* genome had only been published a year earlier in 2002 [2]. The prompt public availability of the sequences allowed the expedient design of 70-bp oligonucleotide arrays corresponding to every gene in the *P*. *falciparum* genome [3]. The investigators leveraged the ability to culture this parasite at scale to prepare RNA at different time points within the IDC for quantitative assessment on an oligonucleotide-based microarray. This approach resulted in the first visualization of the once-a-cycle just-in-time transcription of the majority of genes within the malaria parasite, resembling the cascades of a viral-like life cycle. Strikingly, it provided a catalogue of all the expressed genes in the IDC at a genomic level, a quantum leap in scale beyond the traditional methods of northern blotting and quantitative reverse transcription PCR (qRT-PCR) (Fig 1). The quantitative data obtained in the early days were originally received with some degree of skepticism by the field but have clearly stood the test of time, and this paper [1] is now one of the most highly cited in the field (Google Scholar: 1,844 citations at the time of this publication).

The oligonucleotide arrays were particularly useful for comparing different transcriptomes. The printing of the arrays was inexpensive, making them available for numerous applications. In addition to the fine resolution of the different stages of the IDC, the arrays were used to assess perturbations to in vitro cultures, including the addition of drugs and inhibitors to assess molecular mechanisms [4], and the functional analysis of genetic mutants to identify specific transcriptional programs [5]. Transcriptomics were used to assess expression-level polymorphisms between strains, resulting in the identification of a “variantome,” relevant to virulence gene expression [6]. Finally, hundreds of parasites from endemic populations were interrogated, revealing transcriptional programs associated with distinct transmission strategies [7]. In a parallel approach, the Affymetrix platform was similarly used for the analysis of the *P*. *falciparum* transcriptome over the parasite developmental cycle [8], revealing expression of specific genes at the previously obscure insect and liver stages.

Transcriptomics, trailblazed by these studies in *P*. *falciparum*, has transformed the field of parasitology. The technology has been democratically applied to numerous parasites (other *Plasmodium* spp. and other parasites of medical importance), enabling comparative studies that have revealed species-specific innovations. General challenges have included the need for synchronization, the presence of transcriptionally active host cells, and the short duration of the cell cycle (a major challenge for the evolutionarily related apicomplexan *Toxoplasma gondii*; [9]), as well as the availability of enough material at different stages of the often complex life cycles of parasites. Nevertheless, transcriptomes are now available for all the major pathogens of humans, facilitating numerous studies aimed at understanding the biology of these pathogens and informing translation for therapeutic development.

Transcriptomics itself evolved from microarrays to the use of next-generation RNA sequencing (RNA-Seq) technologies that allowed for the simultaneous quantitation and visualization of coding and noncoding mRNAs in bulk populations of parasites (Fig 1). Most recently, the advent of single-cell approaches, such as scRNA-Seq, scATAC-Seq, and perturb-Seq, are increasingly adapted and utilized for parasitology studies, pushing this field further into the domain of “data-intensive sciences.” Indeed, several recent studies have generated atlases of scRNA-Seq data for a diverse range of parasites and life cycle stages, including for malaria parasites [10]. These invaluable data resources, combined with computational models, provide powerful tools to study gene expression and regulatory events at single-cell resolution.

Critical to the interpretation of the transcriptomic data has been its integration with other ‘omic data sets. For one, the acquisition of the proteomes through the IDC for *P*. *falciparum* clearly demonstrated a lack of concordance between transcripts and proteins [11]. Much effort is currently being dedicated to the description and understanding of posttranscriptional gene regulation. The acquisition of multiomic data at multiple levels will feed into the advent of systems biology approaches to reveal mechanisms in complex host–parasite systems. Of particular note is the VeuPathDB database, an incredible community resource that is the home of genomic data and analysis tools for eukaryotic pathogens and their vectors.

We anticipate a future where the costs of transcriptomic measurements will go down dramatically and accessibility will increase, placing it at the heart of the analysis of parasite biology. Advanced algorithms and software packages have been developed for the analysis, integration, and interpretation of multiomics and single-cell data. However, many challenges remain unresolved. For instance, computational models are primarily developed for mammalian cells, and several challenges limit their utility for the analysis of parasite data. New approaches and tools from statistics, computer science, and data engineering are needed to explicitly model and integrate the unique features of parasite biology. In addition, unlike model organisms, where extensive information is available on regulatory networks, metabolic pathways, and posttranslational modifiers, such resources for parasites are limited. Most genes in parasites are currently functionally uncharacterized, and the parasite genomes are poorly annotated. These limitations hamper the development of holistic systems biology approaches to study cellular processes in parasites. However, new datasets on regulatory interactions are being generated at a rapid pace, such as ChIP-seq [12], enabling the integration of multiple sources of information and network biology approaches for parasitology applications.

The future is very exciting, with advances in new technologies linking ‘omics data directly to the cell and organismic biology of the parasite, such as spatial transcriptomics and imaging mass cytometry. In parallel, advances in the fields of artificial intelligence (AI) and machine learning have revolutionized the analysis of large-scale datasets, significantly impacting science and technology, including fields such as cancer research. Large-scale adaptation of these emerging technologies and AI tools towards parasitology applications in the coming decade will likely result in a major leap forward in our understanding of the basic biology of the parasite and its host. However, translating data into biological insight requires novel and creative approaches in cross-disciplinary collaborative research as well as the training of a new generation of “multilingual” parasitologists with a broad range of skills and knowledge in both data sciences and parasite biology.

## Abbreviations

AI: artificial intelligence
IDC: intraerythrocytic developmental cycle
qRT-PCR: quantitative reverse transcription PCR
RNA-seq: RNA sequencing
